# NFkB hyperactivation causes invasion of esophageal squamous cell carcinoma with EGFR overexpression and p120-catenin down-regulation

**DOI:** 10.18632/oncotarget.24358

**Published:** 2018-01-29

**Authors:** Heather L. Lehman, Michal Kidacki, Joshua I. Warrick, Douglas B. Stairs

**Affiliations:** ^1^ Department of Pathology, The Pennsylvania State University College of Medicine, Hershey, Pennsylvania, USA

**Keywords:** p120-catenin, EGFR, NFkB, RhoA, ESCC invasion

## Abstract

Four out of five patients diagnosed with esophageal squamous cell carcinoma (ESCC) will die within five years. This is primarily a result of the aggressive invasive potential of the disease. Our research is focused on the interplay between tumor suppressors and oncogenes in the invasive process. Specifically, EGFR and p120-catenin (p120ctn) are commonly dysregulated genes that are indicative of poor prognosis in ESCC. In a previous study we demonstrated that in our 3D organotypic culture model, only when EGFR overexpression is combined with p120ctn inactivation do the cells transform and invade – as opposed to either event alone. The purpose of this present study was to identify the components of the molecular pathways downstream of p120ctn and EGFR that lead to invasion. Using both human esophageal keratinocytes and human ESCC cells, we have identified NFkB as a central regulator of the invasive process downstream of p120ctn down-regulation and EGFR overexpression. Interestingly, we found that NFkB is hyperactivated in cells with EGFR overexpression and p120ctn inactivation than with either EGFR or p120ctn alone. Inhibition of this NFkB hyperactivation results in complete loss of invasion, suggesting that NFkB signaling is necessary for invasion in this aggressive cell type. Furthermore, we have identified RhoA and Rho-kinase as upstream regulators of NFkB in this process. We believe the cooperation of p120ctn down-regulation and EGFR overexpression is not only important in the aggressive mechanisms of ESCC but could be broadly applicable to many other cancer types in which p120ctn and EGFR are involved.

## INTRODUCTION

Esophageal squamous cell carcinoma (ESCC) is the most predominant histological subtype of esophageal cancer throughout the world. ESCC remains one of the most aggressive types of cancer, with rapid progression and invasion to local organs. As such, the disease has a dire prognosis with a 5-year overall survival rate around 14% [1]. Furthermore, the majority of patients are diagnosed at an advanced tumor stage, where the 5-year overall survival rate drops to below 5% [1–4]. While modest improvements have been made in diagnostics and therapeutics over the last decade, the molecular mechanisms underlying the aggressive nature of ESCC are yet to be understood.

Like many malignancies, ESCC develops as a result of multiple genetic alterations. Previous studies have highlighted the importance of the tumor suppressor p120-catenin (p120ctn; *CTNND1*) and the oncogene epidermal growth factor receptor (EGFR) in ESCC [5–7] individually. Notably, we demonstrated the importance of p120ctn and EGFR together and that the intersection of these two genetic events (down-regulation of p120ctn and overexpression of EGFR) results in a cell type that morphologically and molecularly mimics the phenotype of invasive human ESCC [5]. The development of the invasive phenotype occurred only when p120ctn was down-regulated and EGFR was overexpressed together, not when either event occurred independently [5].

In our present study we aimed to determine the molecular mechanisms controlling invasion downstream of p120ctn and EGFR. Using genetically modified human esophageal keratinocytes (EPC cells) and human ESCC (TE) cells to assess the impacts of p120ctn down-regulation and EGFR overexpression, our data suggest that NFkB is a major regulator of the resultant invasive phenotype. NFkB is ubiquitously expressed in all mammalian cells and has classically been shown to be involved heavily in regulating the immune system, inflammation, and cellular growth [8, 9]. NFkB has also been shown to be involved in the activation of oncogenes and other factors related to promoting angiogenesis, proliferation, cell transformation, invasion, and metastasis [8–10]. Very little work to date has been done to study NFkB in ESCC. It has been demonstrated that conditional loss of p120ctn in the esophagus leads to activation of NFkB in esophageal tumors [6]. More recently it has been shown that NFkB p65 expression is upregulated in human ESCC tissues compared to paracancerous tissues and associated with advanced clinical stage and lymph node metastasis [11].

p120ctn down-regulation and EGFR overexpression frequently occurs together in ESCC patients. We believe these genetic alterations play a role in causing ESCC to invade rapidly. The central goal of this study was to identify the molecular components downstream of p120ctn down-regulation and EGFR overexpression that are leading to the invasive phenotype of ESCC. By identifying molecular markers that are contributing to this aggressive phenotype, we may be able to identify potential therapeutic targets to study further with hopes of improving the currently grim prognosis of ESCC patients.

## RESULTS

### p120ctn down-regulation and EGFR overexpression results in hyperactivation of NFkB

**Figure 1:**
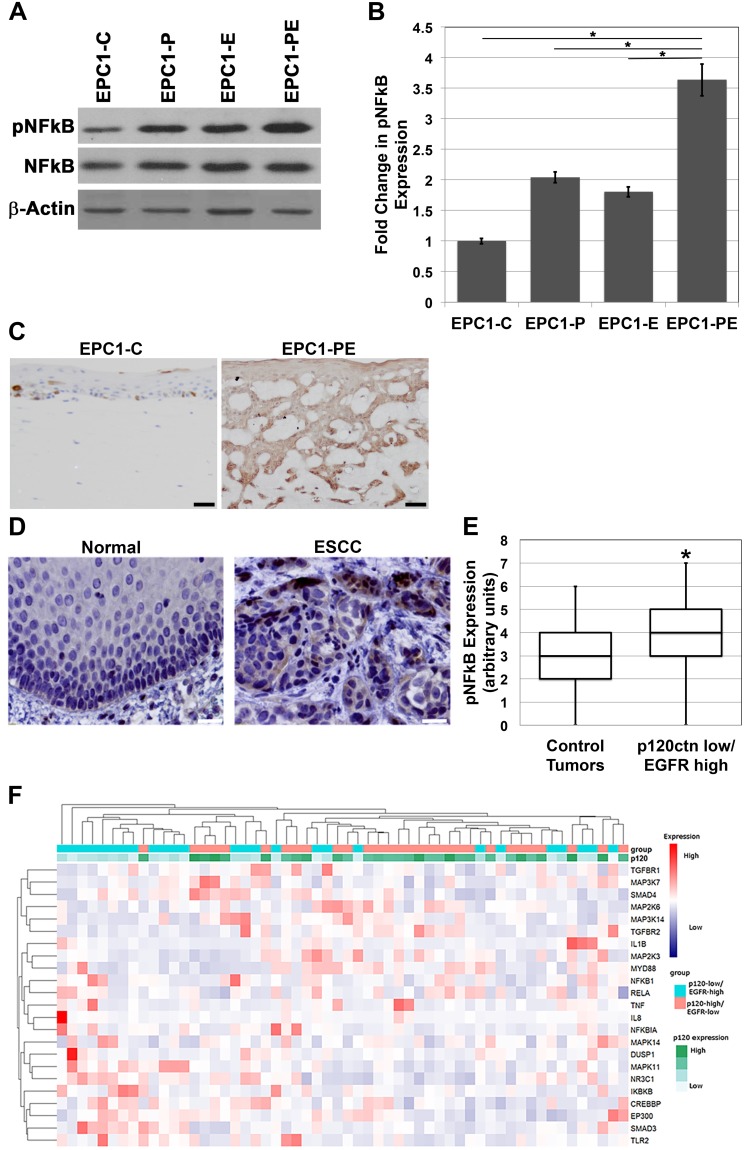
NFkB is hyperactivated in cells with p120ctn down-regulation and EGFR overexpression **(A)** Western blot analysis of EPC1 cells demonstrates increased expression of pNFkB in EPC1-P, -E, and -PE cells compared to EPC1-C control cells, and most significantly in EPC1-PE cells. **(B)** Quantification of pNFkB expression in EPC cells. **(C)** IHC analysis of 3D cultures demonstrates increased pNFkB expression in EPC1-PE cells. **(D)** IHC analysis of human ESCC tissues demonstrates increased pNFkB expression in ESCC when compared to normal adjacent tissue. **(E)** Quantification of IHC demonstrates a significant increase in pNFkB when both p120ctn is down-regulated and EGFR is overexpressed. **(F)** Heat map demonstrates that unsupervised clustering of ESCC tumors using an NFkB gene list separates tumors into two main groups, one enriched with p120ctn-low/EGFR high tumors and the other enriched in p120ctn-high/EGFR low tumors. Scale bar = 50 μm.

p120-catenin and EGFR have been individually shown to lead to downstream activation of NFkB, and thus affect a number of cellular processes associated with tumorigenesis, including invasion [12–17]. Notably, we demonstrated previously that, when p120ctn is down-regulated and EGFR is overexpressed in combination in human esophageal keratinocytes, an invasive cell type results and it closely mimics human ESCC both morphologically and molecularly [5]. To begin to delineate the molecular components involved in creating this invasive cell phenotype downstream of p120ctn and EGFR, EPC human keratinocytes were analyzed for NFkB expression. Western blot analyses show that phosphorylated NFkB (pNFkB; Ser 536) is increased in cells with both p120ctn down-regulation and EGFR overexpression (EPC1-PE) compared to control cells (EPC1-C) or cells with either p120ctn down-regulation (EPC1-P) or EGFR overexpression (EPC1-E) only (Figure 1A and 1B). IHC analyses of the cells in 3D organotypic culture also demonstrate that EPC1-PE cells have increased pNFkB expression (Figure 1C). Interestingly, pNFkB appears to be most strongly expressed in the EPC1-PE cells in the more invasive cells. Furthermore, IHC analysis for pNFkB was performed on 20 human ESCC samples from our own institution and on a purchased tissue microarray of 72 human ESCC samples. These staining show increased pNFkB expression in ESCC tumor tissue when compared to histologically normal-appearing adjacent (control) esophageal epithelia (Figure 1D). Analysis of the quantification of these results demonstrates a significant increase in nuclear pNFkB expression in ESCC tumor samples that had both down-regulated p120ctn and overexpressed EGFR, when compared to control tumor samples (defined as tumors without both genes modified) (Figure 1E). We then broadened our analyses of p120ctn and EGFR in ESCC to include RNA sequencing expression data from 182 human ESCC cases available in The Cancer Genome Atlas database. Unsupervised hierarchical clustering using an NFkB target gene list separated ESCC tumors into two main groups – 1) enriched in p120ctn-low/EGFR-high expression or 2) p120ctn-high/EGFR-low expression (p=0.02, Fisher’s exact test). Twenty-six tumors were identified as being p120ctn-low/EGFR-high, and the heat map in Figure 1F demonstrates increased expression of NFkB and many of its molecular pathway components in this subset of ESCC tumors.

### Inhibition of NFkB activity results in decreased invasion in EPC-PE cells

Our previous study demonstrated that p120ctn down-regulation, together with EGFR overexpression, is a condition that is represented in a large proportion of ESCCs [5]. Furthermore, as shown in Figure 1, pNFkB expression in EPC cells is activated to its highest levels when p120ctn is down-regulated and EGFR is overexpressed together. Therefore, this study is focused on the significant intersection between these two important genes (p120ctn and EGFR) in ESCC. This is reflected in the experimental design in this study, with a focus on examining the condition of p120ctn down-regulation/EGFR overexpression (-PE) compared to the control condition (-C) in our cells.

**Figure 2:**
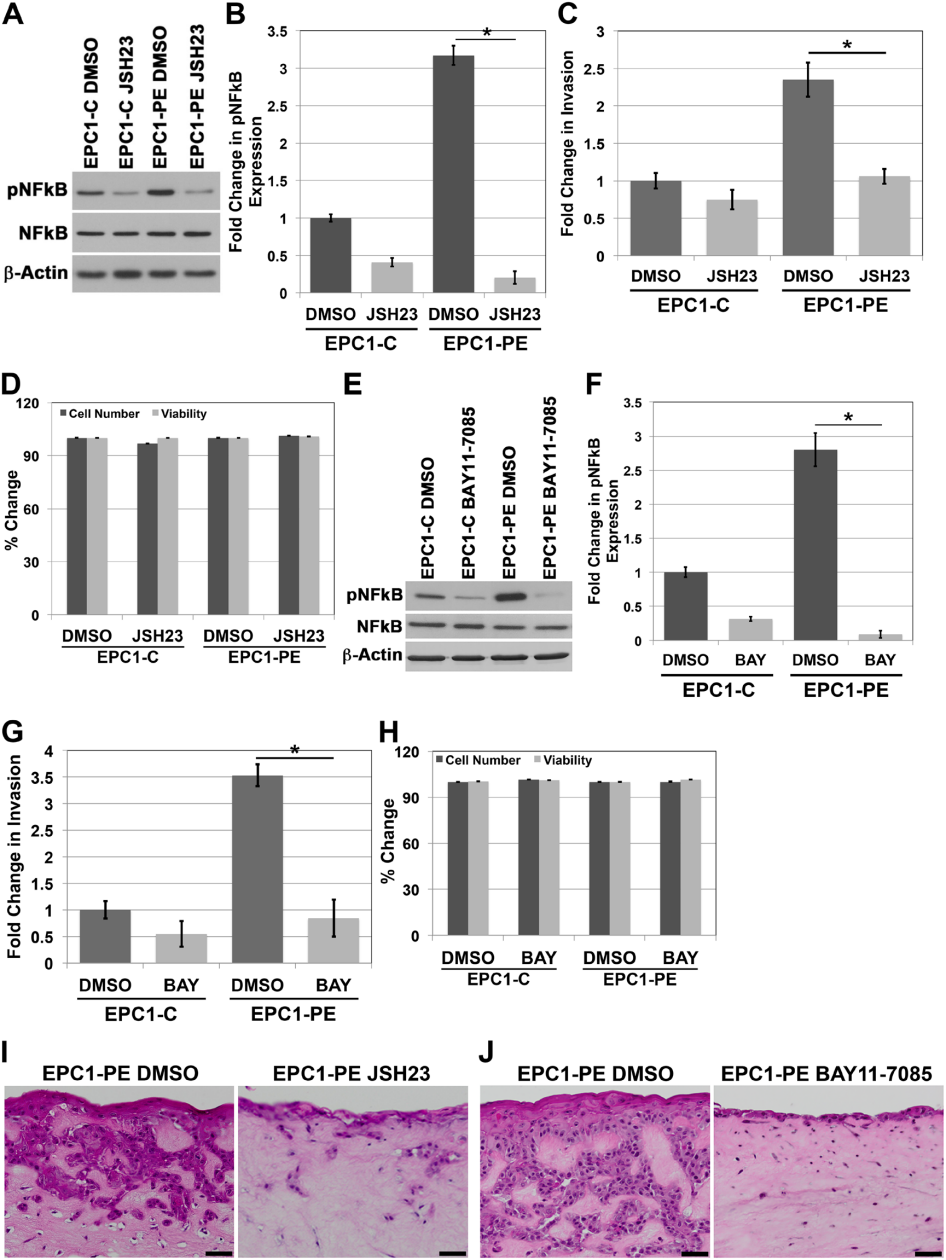
Inhibition of NFkB activity results in decreased invasion of cells with p120ctn down-regulation and EGFR overexpression **(A)** Western blot analysis demonstrates that EPC1-C and EPC1-PE cells treated with JSH-23 have diminished levels of pNFkB. **(B)** Quantification of pNFkB expression with JSH-23. **(C)** *In vitro* invasion assays demonstrate a significant decrease in invasion of EPC1-PE cells when NFkB is inhibited by JSH-23. **(D)** Cell counts and Trypan blue staining demonstrate no changes in cell numbers or cell viability when cells are treated with JSH-23 for 16 hours. **(E)** Western blot analysis demonstrates that EPC1-C and EPC1-PE cells treated with BAY 11-7085 have decreased levels of pNFkB expression. **(F)** Quantification of pNFkB expression with BAY 11-7085. **(G)** *In vitro* invasion assays demonstrate a significant decrease in invasion of EPC1-PE cells when NFkB is inhibited by BAY 11-7085. **(H)** Cell counts and Trypan blue staining demonstrate no changes in cell numbers or cell viability when cells are treated with BAY 11-7085 for 16 hours. **(I)** NFkB inhibition by JSH-23 in EPC1 3D cultures demonstrates inhibition of cellular invasion. **(J)** NFkB inhibition by BAY 11-7085 in EPC1 3D cultures demonstrates inhibition of cellular invasion. Scale bar = 50 μm. ^*^ p<0.05.

Upon examining the increased activation of NFkB when p120ctn is down-regulated and EGFR is overexpressed, we sought to assess whether NFkB was playing a role in the invasive phenotype of the cells. We first used JSH-23 to pharmacologically inhibit NFkB, as this compound selectively inhibits NFkB nuclear translocation, and therefore, activation of NFkB and its transcriptional activity [18]. Using this inhibitor, we were able to successfully knock down pNFkB expression levels below baseline control levels (Figure 2A and 2B). We then examined the *in vitro* invasive capabilities of the EPC1-PE cells using Matrigel invasion assays. Results of these experiments demonstrated a significantly increased ability of EPC1-PE cells to invade compared to EPC1-C control cells when treated with DMSO vehicle control, as expected. However, upon inhibition of NFkB activity with JSH-23, the invasive potential of the EPC1-PE cells was completely inhibited to the level of EPC1-C cells (Figure 2C). To be sure that the inhibitor was not affecting cellular processes such as survival and proliferation, we tested the cells for changes in cell number and viability using cell counts and Trypan blue staining. As shown in Figure 2D, treatment with JSH-23 did not result in changes in cell numbers or cell viability. Therefore, experiments using this inhibitor are not impacted by potential differences in cell number or cell viability. In order to validate these results, we used another NFkB inhibitor, BAY 11-7085, with a different mechanism of action. BAY 11-7085 blocks phosphorylation of IkB-α, thereby selectively and irreversibly inhibits NFkB activation [19, 20]. Similar results are demonstrated with the use of BAY 11-7085 to inhibit pNFkB in EPC1-PE cells (Figure 2E and 2F). The *in vitro* invasive capabilities of EPC1-PE cells upon pNFkB inhibition with BAY 11-7085 were similarly completely inhibited (Figure 2G). As with JSH-23, we tested the cells treated with BAY 11-7085 for changes in cell number and viability using cell counts and Trypan blue staining. Figure 2H shows that BAY 11-7085 does not affect cell numbers or cell viability. Therefore, experiments using this inhibitor are not impacted by potential differences in cell number or cell viability. These experiments were performed and validated using a separate human esophageal keratinocyte cell line, EPC2 cells (Supplementary Figure 1) and yielded identical results. In order to study the importance of NFkB in invasion in a system that even more closely mimics human ESCC, we used EPC1-PE cells in our 3D culture system and inhibited NFkB activity with JSH-23 or BAY 11-7085 treatments. H&E images demonstrate that invasion in 3D culture is inhibited when NFkB activity is inhibited with JSH-23 (Figure 2I) and even more so with BAY 11-7085 (Figure 2J). We also performed NFkB inhibition with JSH-23 and BAY 11-7085 in 3D cultures with EPC1-C cells. H&E images demonstrate that neither NFkB inhibitor affects the thickness of the EPC1-C epithelium (Supplementary Figure 2). Together, these data demonstrate that inhibition of NFkB 1) does not affect EPC1-C epithelium in 3D cultures, 2) does not affect the cell counts of EPC1-PE cells, and 3) results in inhibition of EPC1-PE invasion in Matrigel and 3D cultures. Therefore, these data suggest that NFkB is regulating invasion in an aggressive cell type when p120ctn is down-regulated and EGFR is overexpressed.

### ROCK is involved in controlling NFkB activity

**Figure 3:**
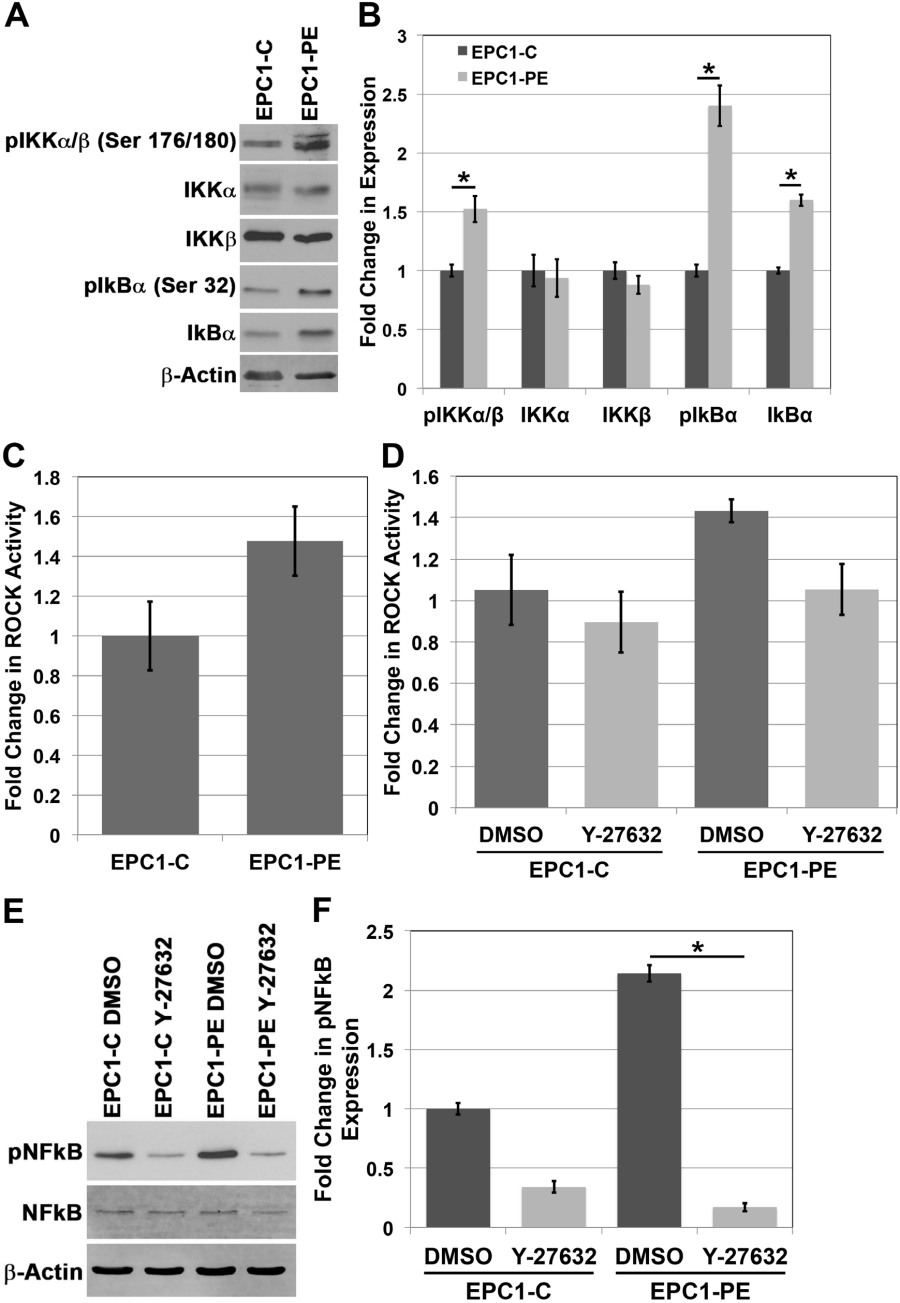
The IKK pathway and Rho-kinase activity play a role in the activation of NFkB **(A)** Western blot analysis of the IKK pathway shows an increase in pIKKα/β and pIkBα expression. **(B)** Quantification of expression of IKK pathway components. **(C)** Colorimetric *in vitro* ROCK activity assay shows an increase in ROCK activity in EPC1-PE cells. **(D)** Inhibition of ROCK activity with Y-27632 ROCK inhibitor. **(E)** Western blot analysis demonstrates inhibition of pNFkB expression in cells treated with Y-27632 ROCK inhibitor. **(F)** Quantification of pNFkB expression with Y-27632.

Given the evident importance of NFkB in regulating the ability of EPC-PE cells to invade, we wanted to further study the molecular components involved in controlling NFkB activity as a result of p120ctn down-regulation and EGFR overexpression. The IkB kinase (IKK) complex is a central component of the NFkB cascade. Typically, NFkB is kept inactive in the cytoplasm by IkB proteins, and NFkB is then activated by IKK-mediated phosphorylation of IkB, causing IkB degradation. This, in effect, results in translocation of NFkB to the nucleus and its activation [21]. Analysis of IKK complex components in EPC cells demonstrates no change in total expression of IKKα or IKKβ between EPC1-C and EPC1-PE cells, though there is an increase in phosphorylated levels of IKKα and IKKβ (Ser 176/180) in EPC1-PE cells (Figure 3A and 3B). Importantly, there is also an increase in pIkBα (Ser 32) expression in EPC1-PE cells compared to EPC1-C cells, suggesting this protein’s involvement in the activation of NFkB in PE cells (Figure 3A and 3B). A similar result is seen in EPC2 cells (Supplementary Figure 3).

Rho-associated protein kinases (ROCK) were the first identified downstream effectors of the small GTP-binding proteins Rho GTPases [22]. The downstream targets of ROCK are not completely clear; however, given their importance downstream of Rho and the previously demonstrated link between Rho and NFkB [23, 24], we decided to investigate if ROCK was playing a role in mediating the activation of NFkB when p120ctn is down-regulated and EGFR is overexpressed. Results of an *in vitro* ROCK activity assay show an increase in ROCK activity levels in EPC1-PE cells compared to EPC1-C cells (p = 0.06) (Figure 3C). The magnitude of the activity seen in this assay is comparable to previously published data [25, 26]. We then wanted to examine the effects of ROCK activity on downstream activation of NFkB in our system. Using a pharmacologic inhibitor, Y-27632, that selectively inhibits ROCK by competing with ATP for binding to its catalytic site, we were able to inhibit ROCK activity in EPC cells (p = 0.069) (Figure 3D). While the changes in ROCK activity are not statistically significant, they appear to be biologically significant, as we analyzed pNFkB expression by Western blot upon inhibition of ROCK and identified a significant decrease in NFkB activation when ROCK activity is inhibited (Figure 3E and 3F). Likewise, similar results were observed in EPC2 cells (Supplementary Figure 3). These data suggest that ROCK is playing a role in mediating NFkB activation downstream of p120ctn down-regulation and EGFR overexpression.

### RhoA is important for NFkB activity and cellular invasion when p120ctn is down-regulated and EGFR is overexpressed

**Figure 4:**
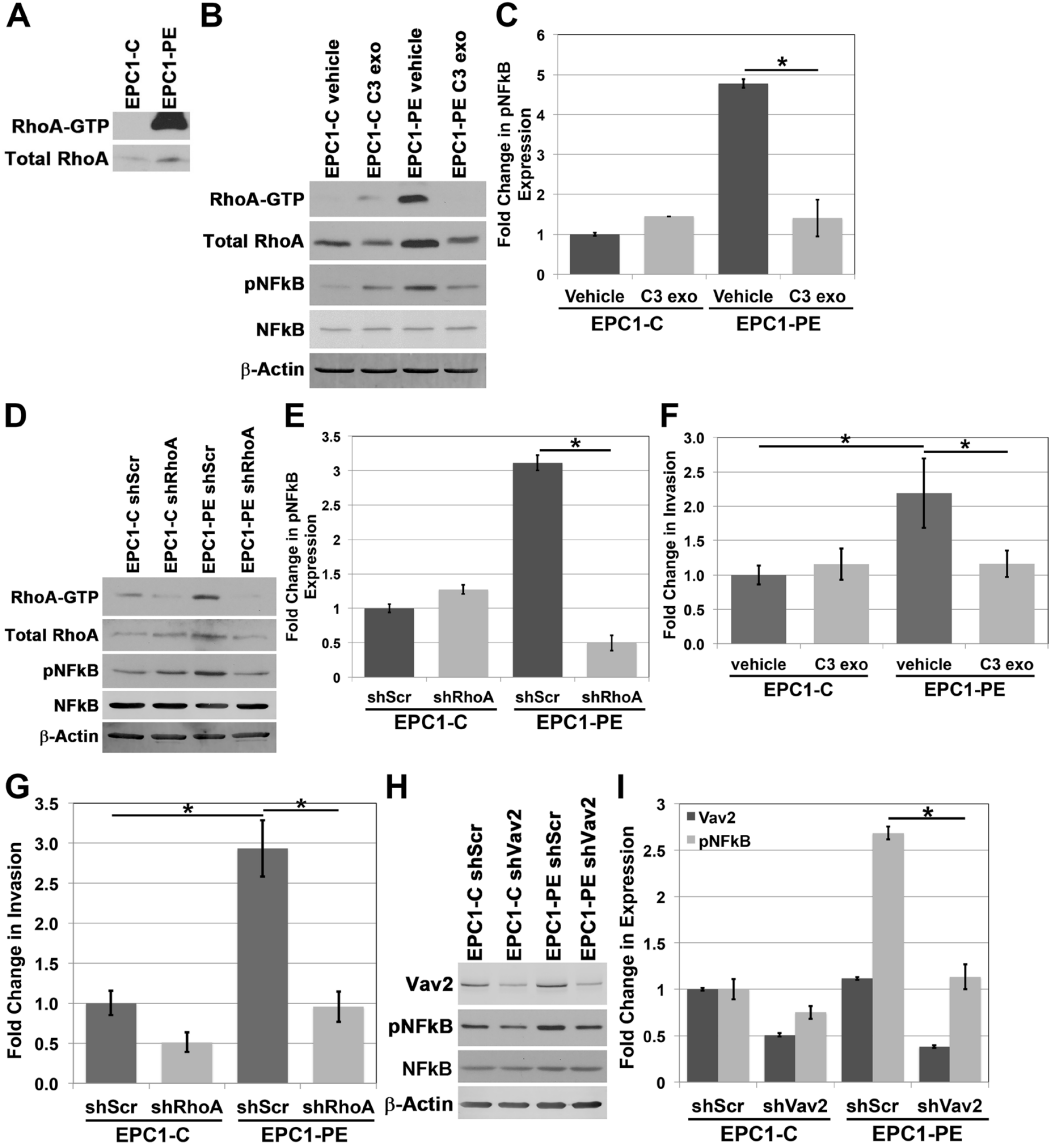
RhoA is involved in regulating NFkB activity **(A)** RhoA pulldown activation assay demonstrates increased RhoA-GTP in EPC1-PE cells. **(B)** Western blot analysis demonstrates inhibition of RhoA-GTP with C3 exotransferase in EPC1-PE cells results in inhibition of pNFkB expression. **(C)** Quantification of pNFkB expression with C3 exotransferase. **(D)** Western blot analysis demonstrates inhibition of RhoA-GTP with shRhoA in EPC1-PE cells results in inhibition of pNFkB expression. **(E)** Quantification of pNFkB expression with RhoA shRNA. **(F)** *In vitro* invasion assays demonstrate a significant decrease in invasion of EPC1-PE cells when RhoA-GTP is inhibited by C3 exotransferase. **(G)** *In vitro* invasion assays demonstrate a significant decrease in invasion of EPC1-PE cells when RhoA-GTP is inhibited by RhoA shRNA. **(H)** Western blot analysis demonstrates inhibition of Vav2 with shVav2 in EPC1-PE cells results in decreased pNFkB expression. **(I)** Quantification of Vav2 and pNFkB expression levels. ^*^ p<0.05.

NFkB and ROCK are known to be downstream of the Rho family of proteins. RhoA is a GTP-binding protein involved in multiple cellular processes, including adhesion, proliferation, cellular movement and cytoskeletal dynamics [27, 28]. Because RhoA is a GTPase and cycles between an active, GTP-bound and an inactive, GDP-bound state, overall expression levels do not reflect the dynamic activity of RhoA [28]. Therefore, we focused our analyses of RhoA on its active, GTP-bound state. Our initial analysis of RhoA-GTP expression in EPC1 cells demonstrates high levels of active RhoA-GTP in EPC1-PE cells where p120ctn is down-regulated and EGFR is overexpressed, compared to almost no detectable expression of RhoA-GTP in control cells (Figure 4A). We then wanted to identify if RhoA was having an effect on the hyperactivation of NFkB we observed in cells with p120ctn down-regulation and EGFR overexpression. To do this we treated EPC1 cells with C3 exotransferase, a widely used pan-Rho inhibitor (inhibiting RhoA, RhoB, and RhoC). Figure 4B and 4C demonstrate that when we inhibit Rho activity in EPC-PE cells, there is a decrease in pNFkB expression. These results were confirmed and extended to RhoA specifically with a second method of inhibition, using a RhoA-specific shRNA. EPC1 cells were nucleofected with shRhoA, and a similar result was demonstrated, showing a decrease in NFkB activity when RhoA is inhibited (Figure 4D and 4E). These experiments were also performed on EPC2 cells and similar results were observed (Supplementary Figure 4). These data suggest that RhoA plays a role in controlling NFkB activity levels.

As demonstrated, EPC cells with p120ctn down-regulation and EGFR overexpression have high levels of cellular invasion compared to control cells. These cells also have high levels of NFkB activity, and when NFkB is inhibited, the cells’ ability to invade is significantly diminished. Given our finding that RhoA is contributing to the control of NFkB activity levels, we wanted to determine whether RhoA has an effect on the invasive capabilities of the cells through NFkB. Therefore, we treated EPC1 cells with either C3 exotransferase or shRhoA and their respective controls, and subsequently performed *in vitro* Matrigel invasion assays. Figure 4F and Figure 4G demonstrate that with either method of inhibition of RhoA activity, the invasive capabilities of EPC1-PE cells are significantly inhibited. Likewise, similar results were observed in EPC2 cells (Supplementary Figure 4). These data suggest that RhoA plays a role in NFkB activity and subsequent invasion of cells with p120ctn down-regulation and EGFR overexpression.

The manner in which RhoA is regulated specifically downstream of p120ctn and EGFR has not yet been studied; however, the activity of small guanine nucleotide-binding proteins like RhoA is regulated by guanine nucleotide exchange factors (GEFs) and GTPase-activating proteins (GAPs) [29]. Vav2 acts as a GEF for Rho family proteins and has been shown to be a potent activator of RhoA [30, 31]. We postulated that Vav2 might have an effect on NFkB activity downstream of RhoA. To examine this, we treated EPC1 cells with Vav2-specific shRNA to inhibit Vav2 expression. Figure 4H demonstrates that when Vav2 expression is decreased through shRNA, pNFkB expression is also decreased in cells with p120ctn-downregulation and EGFR overexpression. Quantification of Vav2 and pNFkB expression is displayed in Figure 4I. These results were confirmed with a second independent clone of shRNA which showed similar results (data not shown), and experiments were performed in EPC2 cells with similar results (Supplementary Figure 4). These data suggest that Vav2 plays a role in the molecular pathway downstream of p120ctn down-regulation and EGFR overexpression leading to NFkB activation.

### Overexpression of NFkB rescues the invasive phenotype after inhibition of RhoA activity

**Figure 5:**
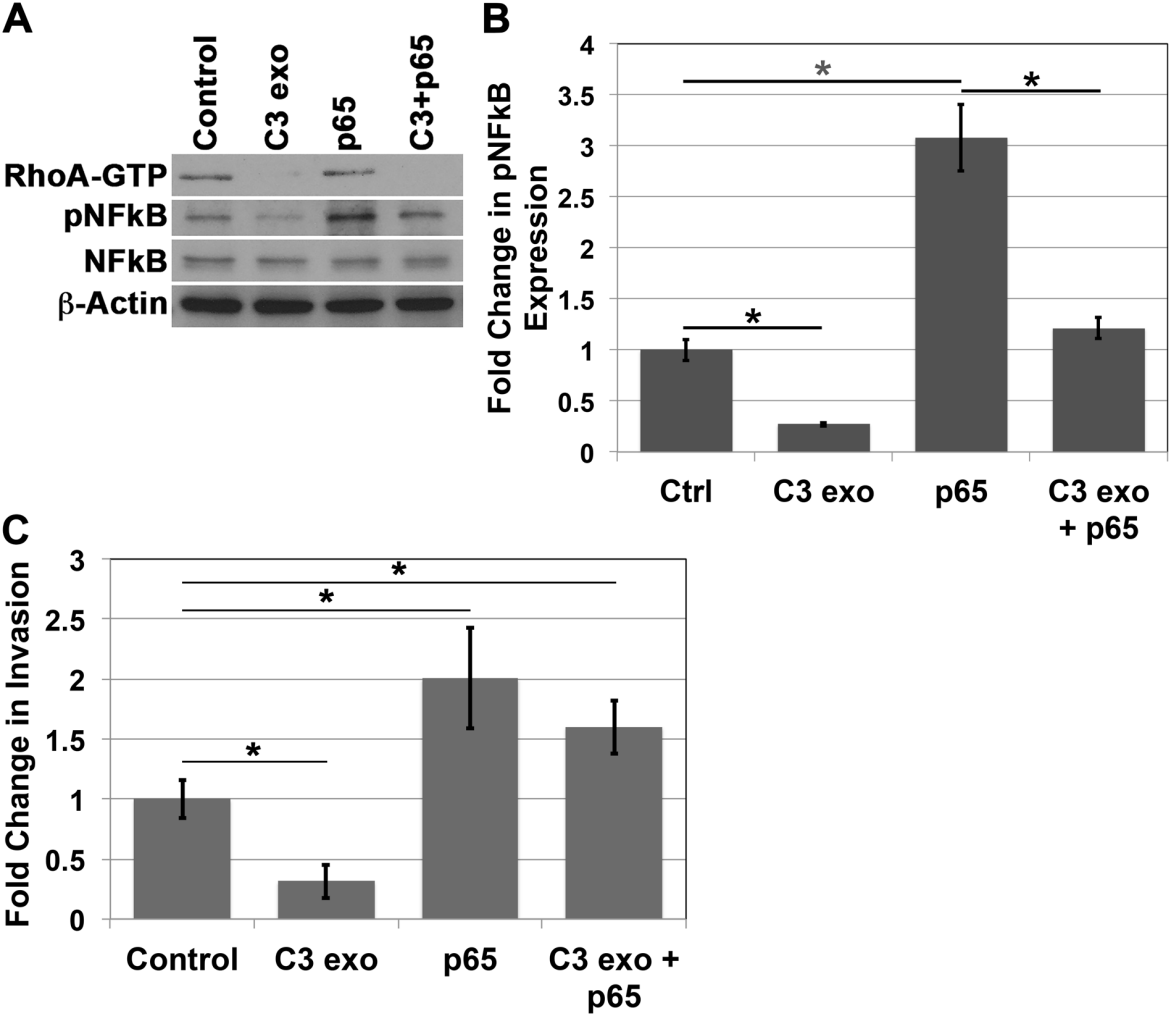
NFkB overexpression rescues invasion after RhoA inhibition **(A)** Western blot analysis demonstrates that treatment of EPC1-PE cells with C3 exotransferase results in inhibition of RhoA and pNFkB, p65 cDNA nucleofection results in increased expression of pNFkB, and C3 exotransferase treatment followed by p65 cDNA nucleofection results in a rescue of pNFkB expression. **(B)** Quantification of pNFkB expression. **(C)** *In vitro* invasion assays demonstrate that treatment of EPC1-PE cells with C3 exotransferase results in inhibition of invasion, p65 cDNA nucleofection results in increased invasion, and C3 exotransferase treatment followed by p65 cDNA nucleofection results in a rescue of the invasive phenotype. ^*^ p<0.05.

In order to be thorough in our analysis of the role of NFkB in invasion when p120ctn is down-regulated and EGFR is overexpressed in EPC cells, we performed a rescue experiment. Here we treated EPC1-PE cells with C3 exotransferase to inhibit RhoA activity, followed by nucleofection of the cells with p65 cDNA. Western blot analyses demonstrate inhibition of RhoA-GTP upon treatment with C3 exotransferase, as well as overexpression of pNFkB upon independent nucleofection with p65 cDNA. Importantly, when EPC1-PE cells were treated with C3 exotransferase followed by p65 cDNA, pNFkB phosphorylation levels were restored back to expected levels similar to that of the cells treated with vehicle controls (Figure 5A and 5B). Even more interesting were the results of the subsequent *in vitro* Matrigel invasion assays. These experiments demonstrated that, as expected, inhibition of RhoA caused a decrease in invasion and overexpression of pNFkB resulted in an increase in invasion of the cells. Notably, in EPC1-PE cells with RhoA inhibition followed by overexpression of pNFkB, the invasive phenotype was rescued and reached levels similar to that of EPC1-PE cells treated with vehicle controls (Figure 5C). These experiments were performed in EPC2 cells with similar results as well (Supplementary Figure 5). These data suggest that NFkB is indeed playing a major regulatory role in the invasive process when p120ctn is down-regulated and EGFR is overexpressed in cells that mimic human ESCC.

### NFkB regulates invasion in human ESCC cells

**Figure 6:**
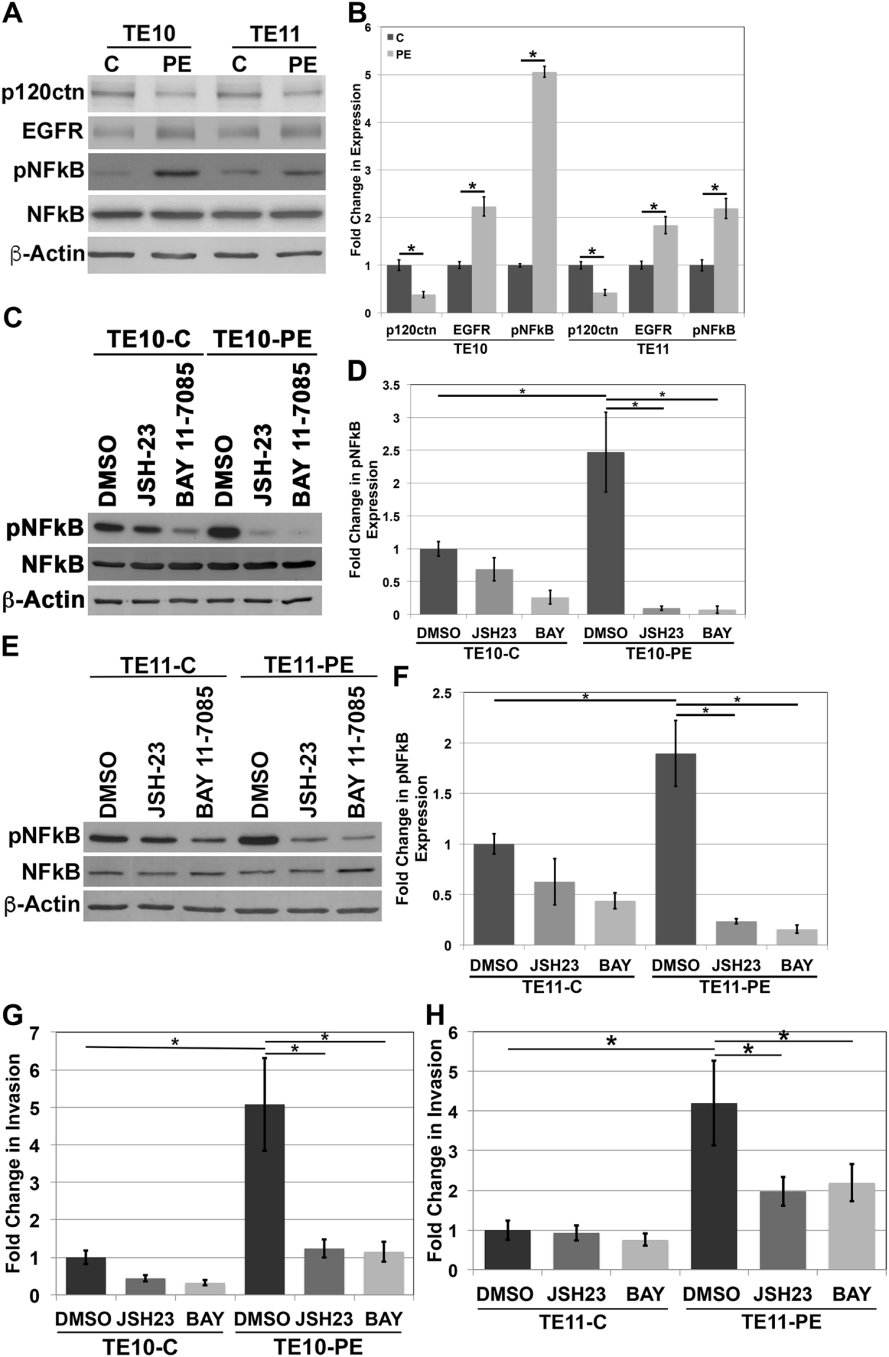
NFkB regulates invasion in human ESCC cells **(A)** Western blot analysis of TE10 and TE11 cells demonstrates an increase in pNFkB expression when p120ctn is down-regulated and EGFR is overexpressed. **(B)** Quantification of p120ctn and EGFR expression in TE10-C and -PE and TE11-C and -PE cells. **(C)** Western blot analysis demonstrates inhibition of pNFkB when TE10 cells are treated with JSH-23 or BAY 11-7085. **(D)** Quantification of pNFkB expression in TE10 cells treated with JSH-23 or BAY 11-7085. **(E)** Western blot analysis demonstrates inhibition of pNFkB when TE11 cells are treated with JSH-23 or BAY 11-7085. **(F)** Quantification of pNFkB expression in TE11 cells treated with JSH-23 or BAY 11-7085. **(G)** *In vitro* invasion assays demonstrate that inhibition of pNFkB with either JSH-23 or BAY 11-7085 results in a significantly decreased ability of TE10-PE cells to invade. **(H)** *In vitro* invasion assays demonstrate that inhibition of pNFkB with either JSH-23 or BAY 11-7085 results in a significantly decreased ability of TE11-PE cells to invade. ^*^ p<0.05.

We have demonstrated that the EPC human esophageal keratinocytes used in our studies both molecularly and phenotypically mimic human ESCC [5]. We wanted to further the relevance of our study by examining NFkB levels in human ESCC cell lines. We examined TE10 and TE11 cells, both widely used in *in vitro* studies of ESCC [32–36]. We down-regulated p120ctn and overexpressed EGFR in both cell lines through nucleofection with both a p120ctn shRNA and EGFR Del overexpression vector (called TE10-PE and TE11-PE). Subsequent Western blot analyses of protein expression in TE10 and TE11 cell lines demonstrates knock down of p120ctn expression and increased EGFR expression in TE10-PE and TE11-PE cells (Figure 6A). Quantification of the changes in p120ctn and EGFR expression in TE10 and T11 cells is displayed in Figure 6B. Importantly, the ESCC cells with down-regulated p120ctn and overexpressed EGFR had increased expression levels of pNFkB (Figure 6A).

To determine whether NFkB is playing a role in the invasive phenotype of ESCC cells, NFkB was inhibited in TE10 and TE11 cells through treatment with either JSH-23 or BAY 11-7085, as described previously. Following treatment, cells were placed in Matrigel invasion assays. Figure 6C and 6D demonstrate inhibition of pNFkB expression levels through pharmacologic treatment of TE10-C and TE10-PE cells. Similarly, Figure 6E and 6F demonstrate inhibition of pNFkB expression through JSH-23 or BAY 11-7085 treatments in TE11-C and TE11-PE cells. Matrigel invasion assays demonstrated that TE10 and TE11 cells with p120ctn down-regulation and EGFR overexpression have significantly increased invasive capabilities when compared to their controls (Figure 6G and 6H). This suggests that hyperactivated NFkB as a result of p120ctn down-regulation and EGFR overexpression may lead to a more invasive phenotype. Most notably, we found that inhibition of NFkB activity in these ESCC cells resulted in inhibition of the ability of the TE10-PE and TE11-PE cells to invade (Figure 6G and 6H). These data strongly support our earlier findings in our transformed human esophageal keratinocytes that mimic ESCC and more substantially suggest that NFkB regulates ESCC invasion when p120ctn is down-regulated and EGFR is overexpressed.

## DISCUSSION

Down-regulation or loss of p120ctn has been demonstrated in ESCC as well as eleven other cancer types [6, 37–47]. Similarly, overexpression of EGFR has been implicated as significant in these cancer types and various others [48–52]. Cancers frequently develop in the context of multiple genetic modifications. Our studies are the first to examine the intersection of p120ctn down-regulation and EGFR overexpression, though we hypothesize that these genetic alterations most likely traverse in many cancer types in addition to ESCC. Therefore, the applicability of our findings may be broad and significantly impact a wide spectrum of cancer and diseases.

We have identified NFkB as a central regulator of invasion in esophageal squamous cell carcinoma when p120ctn is down-regulated and EGFR is overexpressed. Specifically, we demonstrated high levels of NFkB phosphorylation and activation when p120ctn and EGFR are altered together, and this NFkB activity appears to be regulating the invasive capabilities of the cells. NFkB has been shown to be constitutively active in many cell types; though, the mechanisms and reasons for its constitutive activation are not well understood [9, 53–57]. Our data suggest that the synergistic relationship between p120ctn down-regulation and EGFR overexpression is causing NFkB activation, and this may be a possible, though not yet explored, explanation for the hyperactivation of NFkB in other cell types. One contributing factor may be the down-regulation of E-cadherin. p120ctn interacts with and stabilizes E-cadherin at the adherens junction complex. We have demonstrated previously that E-cadherin is down-regulated upon p120ctn down-regulation. Interestingly, we noted even further suppression of E-cadherin levels when both p120ctn was down-regulated and EGFR was overexpressed [5]. In a study examining human ESCC tissues and paracancerous tissues, it was shown that there was a significant decrease in E-cadherin expression in ESCC samples, as well as a significant increase in pNFkB expression in ESCC [11]. pNFkB expression was also significantly correlated with clinical staging, lymph node metastasis, and tumor differentiation [11]. While this study demonstrates a correlation between the loss of E-cadherin and the increased expression of active pNFkB, our present study brings to light a potential mechanism of regulation of both E-cadherin and pNFkB in ESCC.

p120ctn and EGFR have each been shown to individually regulate RhoA GTPase. p120ctn has been shown to inhibit RhoA activation [58], while EGFR can be an activator of RhoA [59]. Therefore, our observations of increased RhoA activity upon down-regulation of p120ctn and overexpression of EGFR confirm these previous findings [60]. Furthermore, RhoA has been shown to play a role in both positive and negative regulation of NFkB depending on the context [23, 60–62]. However, to our knowledge, this present work is one of very few studies to look at RhoA and NFkB in the context of cellular invasion, and no other work has been done on the interaction of these genes in ESCC. Both RhoA and NFkB are involved in the regulation of genes involved in diverse processes in cancer including invasion and metastasis [63–69]. We have identified both RhoA and NFkB to be activated at significantly higher levels when p120ctn is down-regulated and EGFR is overexpressed. These upstream triggers of RhoA-NFkB signaling appear to increase the invasiveness of cells, and could be potential targets for ESCC therapeutics.

It is apparent that NFkB and the signaling pathways that control it, as well as the signaling pathways it controls, are important in a multitude of aspects of cancer. Our data in this study further support this notion. This makes NFkB an attractive target for pharmacologic intervention. Currently, directly targeting NFkB is a challenge in cancer therapy [70, 71]. Much effort has been put forth by pharmaceutical companies to develop multiple types of NFkB inhibitors [72, 73]. As mentioned, NFkB is required for immune functions in the body, and long-term and systematic immune suppression could have detrimental effects on a patient. NFkB inhibition may need to be developed in a way that would avoid long-term immunosuppression.

Another challenge to the use of NFkB inhibitors as therapeutic agents is the need to identify the molecularly defined subset of patients who will preferentially respond to NFkB inhibition. We believe we have laid the groundwork for identifying a subset of ESCC patients (with p120ctn down-regulation and EGFR overexpression together) that may respond to more targeted therapy as a result of the modification of these two genes. In addition, we have identified molecular signaling components that are playing a role in regulating NFkB downstream of p120ctn down-regulation and EGFR overexpression. These components, including RhoA, could possibly be targeted in a specific way that would inhibit NFkB but also minimize system toxicity and avoid innate immunity suppression. In an effort to move relevant ESCC research in this direction, we need to expand our studies to include examining NFkB and its upstream triggers in human ESCC tissue samples as well as *in vivo* models of ESCC with p120ctn down-regulation and EGFR overexpression.

Overall, we have demonstrated that NFkB regulates invasion in ESCC when p120ctn is down-regulated and EGFR is overexpressed. It is hyperactivated downstream of these genetic alterations through increased activity of RhoA GTPase and Rho-kinase. Determination of these pathway components will be useful in future studies aiming to find therapeutic targets for ESCC patients.

## MATERIALS AND METHODS

### Cell lines

EPC1-hTERT and EPC2-hTERT human esophageal keratinocytes were a generous gift from Anil K. Rustgi (University of Pennsylvania, Philadelphia, PA) and were cultivated in keratinocyte serum-free medium, as previously described [5, 74]. Modifications of EPC1 and EPC2 cells were made with TRIPZ and pLVX vectors (Clontech Laboratories, Inc.) to down-regulate p120ctn and overexpress EGFR, as previously described [5]. All derivatives of parental cells will be termed EPC1-(genetic modification) or EPC2-(genetic modification), respectively. EPC1-C and EPC2-C keratinocytes were generated by infection of parental EPC-hTERT cells with a pLVX-IRES-Neo vector and a TRIPZ lentiviral scrambled shRNA, resulting in no change in p120ctn and EGFR expression. As previously described, EPC1-P and EPC2-P cells have down-regulated p120ctn expression, EPC1-E and EPC2-E cells have overexpression of EGFR, and EPC1-PE and EPC2-PE cells have both p120ctn down-regulation and EGFR overexpression [5]. Human fetal esophageal fibroblasts (FEF-SC) were purchased from ScienCell Research Laboratories, Inc. and were grown in Dulbecco’s modified Eagle’s medium (DMEM) (Mediatech, Inc.) supplemented with 10% fetal bovine serum (FBS) (VWR) and 1% penicillin/streptomycin (Invitrogen). TE10 and TE11 human esophageal squamous cancer cell lines were also a generous gift from Anil K. Rustgi and were cultivated in DMEM supplemented with 10% FBS (VWR) and 1% penicillin/streptomycin (Invitrogen). All cell lines were cultivated at 37°C, 5% CO_2_.

### Western blot analysis

Cells were harvested by trypsinization and centrifuged at 1,000 rpm for 5 minutes. Cell pellets were washed in 1X PBS and centrifuged at 2,000 rpm for 5 minutes. Cells were then incubated in lysis buffer as previously described [5]. Protein concentrations were determined using a Coomassie Protein Assay Kit (Pierce Biotechnology), proteins were denatured, and Western blotting was performed as previously described [5]. Western blots were quantified by densitometry using ImageJ software (National Institutes of Health) and normalized to β-Actin loading controls.

### Invasion assays

Invasion assays were performed using BD Biocoat Matrigel Invasion Chambers with 8-μm pore filters (BD Biosciences). The bottom wells of the companion plate were filled with 20% serum-containing medium to act as a chemoattractant for the cells. Cells were seeded at a density of 2.5 x 10^4^ per insert in serum-free medium. Invasion assays were incubated for approximately 16 hours at 37°C, 5% CO_2_, stained with a Diff-Quick stain set (Fisher Scientific) and imaged with an Olympus BX53 light microscope (Olympus America). Invasive cells were counted in five representative high-power fields and all experiments were performed three times.

For invasion experiments involving cells treated with inhibitors, cells were pretreated with the respective dose of inhibitor two hours prior to being harvested for the assay. Inhibitors and vehicle controls were also added to the serum-free medium cell suspension prior to addition into the invasion chambers.

### 3D organotypic culture

EPC cells were grown on a 3D matrix as previously described [5, 75] with the following modifications. The collagen/Matrigel layer had 1.5x10^5^ human fetal esophageal fibroblasts (FEF-SC cells) embedded within it. On day 13, cultures were raised to an air-liquid interface and cultured for four days in Epidermalization III medium, with medium changed every other day. This medium is identical to Epidermalization I medium except that it does not contain progesterone and 2% unchelated newborn calf serum is added. Cultures were grown for a total of 17 days and harvested by fixing half of the culture in 10% neutral buffered formalin (Fisher Scientific), and paraffin-embedding, and peeling epithelium from the other half to process for protein collection.

For 3D cultures treated with BAY 11-7085 or JSH-23, cells were first pretreated for 2 hours with 10 μM of inhibitor prior to being added to the culture on day seven. Then, the inhibitors were added to the 3D culture medium beginning on day nine. 10 μM BAY 11-7085, 10 μM JSH-23, or DMSO was added to the Epidermalization II culture medium. BAY 11-7085, JSH-23, or DMSO was also added during each subsequent medium change until cultures were harvested on day 17 of the assay.

### Rhoa pull-down activation assay

RhoA activity was assessed using the Rho Activation Assay Biochem Kit from Cytoskeleton, Inc. (#BK036) per the manufacturer’s instructions. EPC cells were lysed in 150 μL of ice-cold Cell Lysis Buffer, spun at 10,000 x g for 1 minute at 4°C, and snap frozen in liquid nitrogen. 150 μg of protein was incubated and rotated with 50 μg of rhotekin-RBD beads overnight at 4°C. The rhotekin-RBD beads were pelleted by centrifugation and 2X Laemmli sample buffer was added prior to heating the samples for 2 minutes at 95°C. 20% input cell lysates were included to detect total RhoA. Samples were analyzed by SDS-PAGE and Western blot analysis was performed as previously described [5].

### Rho-associated kinase (ROCK) activity assay

Active ROCK was determined using the Rho-associated Kinase Activity Assay from Millipore (#CSA001) per the manufacturer’s instructions. EPC cell protein lysates were added to MYPT1 pre-coated wells and incubated for 30 minutes at 30°C. Anti-pMYPT1 (Thr696) antibody was added for 1 hour at room temperature, followed by HRP conjugated goat anti-rabbit IgG secondary antibody for 1 hour at room temperature. Substrate Reagent was added to each well followed by Stop Solution (both provided in the assay kit) and absorbance was measured at 450 nm.

### Cell counts and viability

Cell counts and viability assays were performed prior to and following inhibitor treatments. Cells were harvested by trypsinization and centrifuging at 1,000 rpm for 5 minutes. Cells were then stained with Trypan blue dye and counted in the Countess Cell Counter (Invitrogen). Counts and viability of EPC cells treated with inhibitors were then compared to their respective treatment controls (DMSO) and cell line controls.

### Antibodies, inhibitors, and plasmids

The p120ctn antibody (#610134) was purchased from BD Transduction Laboratories and used at 1:10,000 for immunoblotting. Antibodies for EGFR (1:5000; #2232), NFkB (1:1000; #4764), pNFkB (1:1000; #3033), pIKKα/β (1:1000; #2697), IKKα (1:1000; #2682), IKKβ (1:1000; #2678), IkBα (1:1000; #9242), pIkBα (1:1000; #2859) were purchased from Cell Signaling Technology. The antibody against Vav2 (1:5000; ab52640) was from Abcam. The antibody against RhoA (1:500; #ARH03) was from Cytoskeleton, Inc. β-actin (1:10,000; #A5316), used as a loading control for immunoblotting, was purchased from Sigma-Aldrich Corp.

Inhibitors of NFkB and ROCK were obtained from Millipore. Inhibition of NFkB was performed using NFkB Activation Inhibitor II, JSH-23 (#481408) at a concentration of 10 μM for 2 hours or BAY 11-7085 (#196872) at a concentration of 10 μM for 2 hours. Inhibition of ROCK was performed using Y-27632 (#SCM075) at a concentration of 10 μM for 2 hours. Chemical inhibition of RhoA was performed using Rho Inhibitor I (#CT04) from Cytoskeleton, Inc. at a concentration of 0.5 μg/ml for 4 hours.

EPC cells were nucleofected with the following plasmid constructs obtained from GE Dharmacon: human pGIPZ shVav2, clone V2LHS_171903; human pGIPZ shVav2, clone V2LHS_262378; human pGIPZ shRhoA, clone V2LHS_132703; human pGIPZ shScramble (control shRNA). EPC cells were also nucleofected with pcDNA3-p65 (gracious gift from Sankar Ghosh, Columbia University) or pcDNA3 (empty vector; gracious gift from Nicholas Buchkovich, Penn State College of Medicine). TE cells were nucleofected with the following plasmid constructs: pLVX hp120 shRNA; pLVX EGFR Del (746-750); pLVX-IRES-Neo (control plasmid; Clontech Laboratories, Inc.).

### Patients and specimens

Approval was obtained from the Institutional Review Board of Pennsylvania State Hershey Medical Center for all human sample experiments performed in this study. The Surgical Pathology Archive of the Pennsylvania State Hershey Medical Center was searched for ESCC cases. A total of 20 paraffin-embedded human ESCC cases with normal-appearing adjacent epithelium between 1978 and 2013 were selected for inclusion in this study. Ten human normal esophageal epithelium samples were used as controls. A hematoxylin and eosin (H&E)–stained section of each case was examined by an anatomical pathologist to confirm the diagnosis.

Human paraffin-embedded tissue microarrays were purchased from US Biomax, Inc. (HEso-Squ180Sur-01) and stained for p120ctn, EGFR, and pNFkB as described below. Immunoreactivity of TMA samples was scored by an anatomical pathologist.

### Immunohistochemistry

Paraffin sections of 3D organotypic cultures or ESCC tumors were baked for one hour at 60°C and deparaffinized in xylene. Sections were then rehydrated in 100%, 95%, 70% ethanol and distilled water. Antigen unmasking was performed with the Retriever (Electron Microscopy Sciences) in 10 mM sodium citrate buffer, pH 6. Endogenous peroxidases were quenched in 3% hydrogen peroxide for 6 minutes prior to sections being incubated for 30 minutes with a blocking solution of 5% BSA in PBS. Sections were incubated with primary antibodies overnight at 4°C followed by 2 hours with ImmPRESS HRP reagent (Vector Laboratories). HRP activity was then detected using DAB substrate (Thermo Scientific). Sections were dehydrated in 70%, 95%, and 100% ethanol and xylene, and coverslipped with Permount (Fisher Scientific). Sections were imaged with an Olympus BX53 light microscope (Olympus America).

Scoring of IHC was performed using a semi-quantitative system. Immunohistochemical stains were quantified using a variation of the Allred scoring system [76], as previously described [77]. In short, tumor cells were assigned intensity and areas scores for each antibody (nuclear expression NFkB, cytoplasmic p120ctn, and EGFR). Intensity was scored 0 (no staining) to 3 (high intensity staining). Area was scored as 0 (no staining), 1 (approximately 1% cells positive), 2 (approximately 10% cells positive), 3 (approximately one third cells positive), 4 (approximately two thirds cells positive), or 5 (approximately 100% cells positive). Intensity and area scores were added to give a sum ranging from 0 to 8.

### Nucleofection

All transfections were done using a Nucleofector II device and the Nucleofector V kit (Amaxa Biosystems). EPC cells were transfected using program T-030 and TE cells were transfected using program X-001. 10^6^ cells were transfected per reaction in 100 μL of nucleofection buffer with the addition of 1 μg of shRNA. Cells were collected 48 hours post-nucleofection for Western blot and invasion assay analysis.

### Statistical analysis

Comparisons between two groups were performed using Student’s t-tests and a one-way ANOVA was used for multiple comparisons. *P* ≤ 0.05 was considered statistically significant.

### The cancer genome atlas (TCGA) analysis

Normalized RNA sequencing expression data from 182 esophageal squamous cell carcinomas were downloaded from The Cancer Genome Atlas (TCGA) Data Commons (https://portal.gdc.cancer.gov/) using the TCGAbiolinks package [78] using R version 3.3.1 [79]. Expression data were log2 transformed. Based on review of histograms, cases were considered p120ctn-high or p120ctn-low if levels of p120ctn were in the upper or lower quartile, respectively. Cases were considered EGFR-high if expression was greater than the 50^th^ percentile, and EGFR-low otherwise. Unsupervised hierarchical cluster was performed on p120ctn-high/EGFR-low and p120ctn-low/EGFR-high cases [80], using an NFkB gene list from Biocarta (https://cgap.nci.nih.gov/Genes/PathGeneQuery?PAGE=1&ORG=Hs&PATH=h_nthiPathway).

## SUPPLEMENTARY MATERIALS FIGURES



## Abbreviations

DMEM: Dulbecco’s modified Eagle’s medium
DMSO: Dimethyl sulfoxide
EGFR: Epidermal growth factor receptor
ESCC: Esophageal squamous cell carcinoma
FBS: Fetal bovine serum
IHC: Immunohistochemistry
IKK: IkB kinase
NFkB: Nuclear factor kappa-light-chain-enhancer of activated B cells
p120ctn: p120-catenin
D1: Catenin
pNFkB: Phosphorylated nuclear factor kappa-light-chain-enhancer of activated B cells
RhoA: RhoA GTPase
ROCK: Rho-kinase
TMA: Tissue microarray
