# Supplementary material for: NFkB hyperactivation causes invasion of esophageal squamous cell carcinoma with EGFR overexpression and p120-catenin down-regulation
Source: Oncotarget. 2018 Jan 29;9(13):11180–96. doi: 10.18632/oncotarget.24358 (PMC5834278; doi:10.18632/oncotarget.24358)
Supplement: Supplementary file 1 [file oncotarget-09-11180-s001.pdf]

NFkB hyperactivation causes invasion of esophageal squamous cell carcinoma with EGFR overexpression and p120-catenin down-regulation

SUPPLEMENTARY MATERIALS

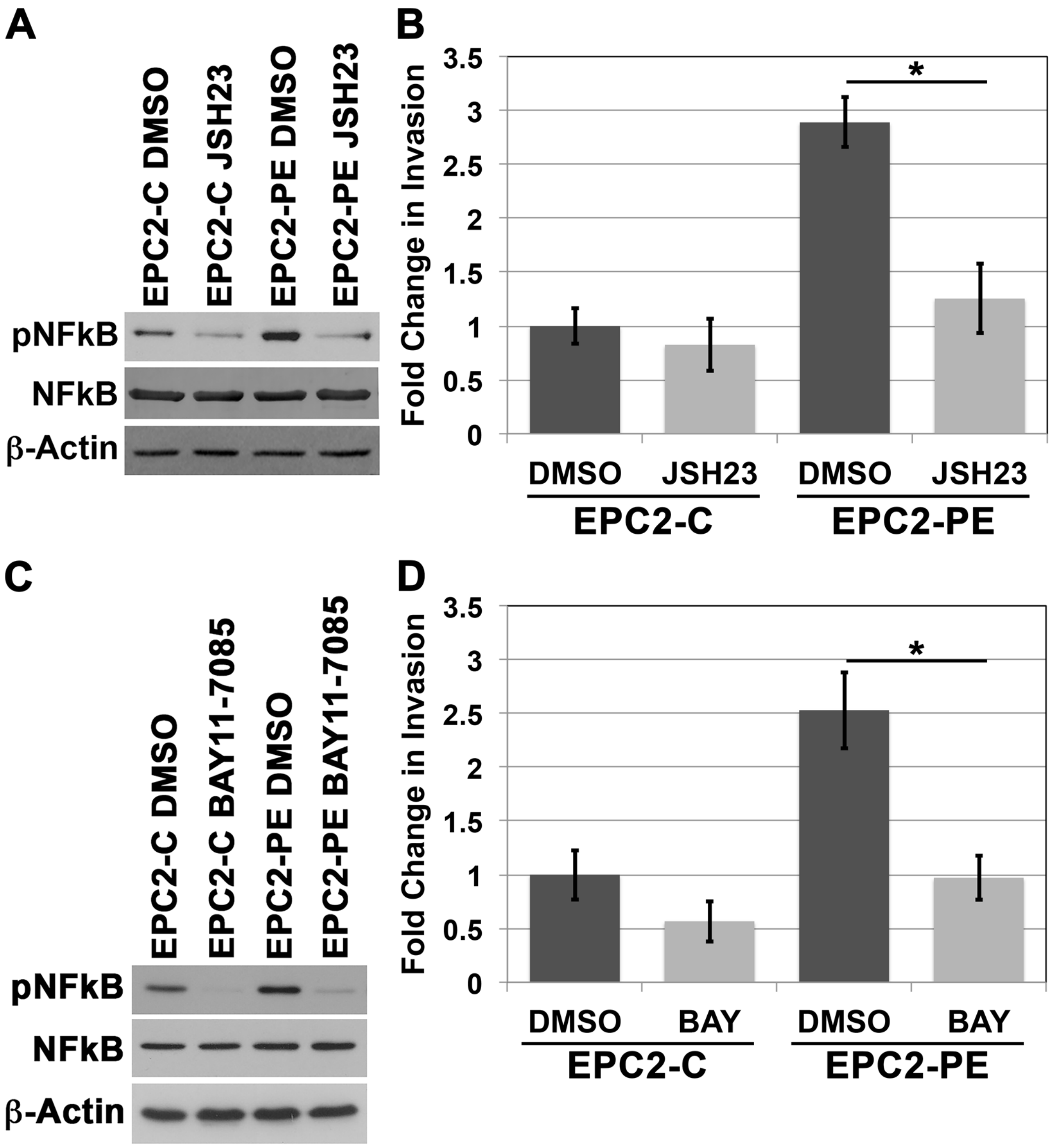

Supplementary Figure 1: Inhibition of NFkB activity results in decreased invasion of cells with p120ctn down-regulation and EGFR overexpression. (A) Western blot analysis demonstrates that EPC2-C and EPC2-PE cells treated with JSH-23 have diminished levels of pNFkB. **(B)** *In vitro* invasion assays demonstrate a significant decrease in invasion of EPC2-PE cells when NFkB is inhibited by JSH-23. **(C)** Western blot analysis demonstrates that EPC2-C and EPC2-PE cells treated with BAY 11-7085 have decreased levels of pNFkB expression. **(D)** *In vitro* invasion assays demonstrate a significant decrease in invasion of EPC2-PE cells when NFkB is inhibited by BAY 11-7085. * $p<0.05$.

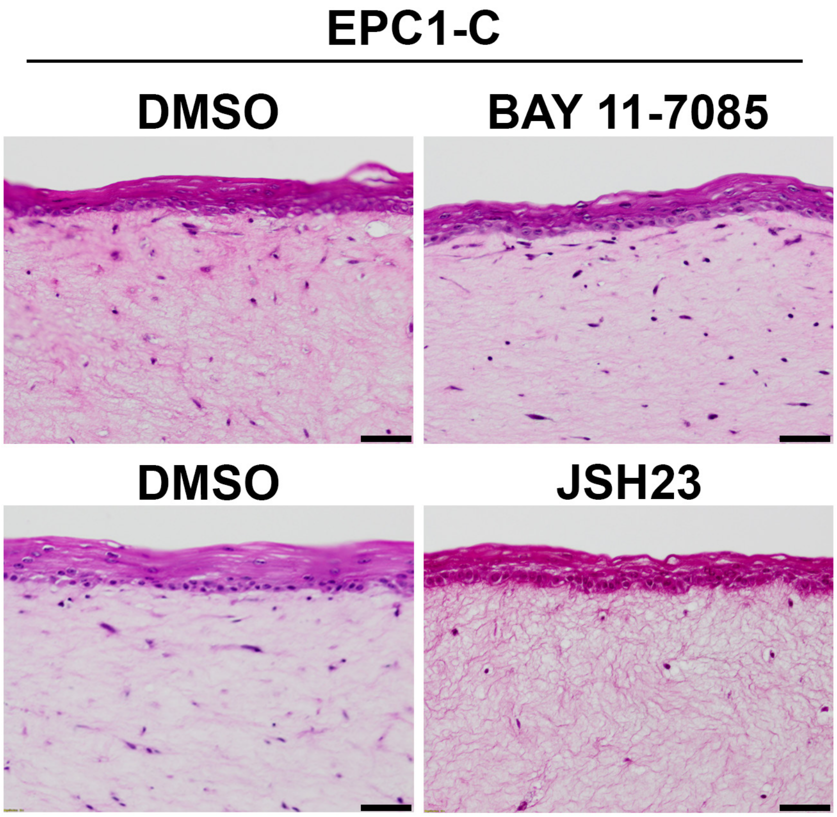

Supplementary Figure 2: Inhibition of NFkB does not affect epithelium thickness in EPC1-C 3D cultures. 3D cultures using control EPC1-C cells demonstrate no significant change in thickness of epithelium in cultures treated with either JSH-23 BAY 11-7085 NFkB inhibitors or DMSO as a vehicle control. Scale bars = 50 μM.

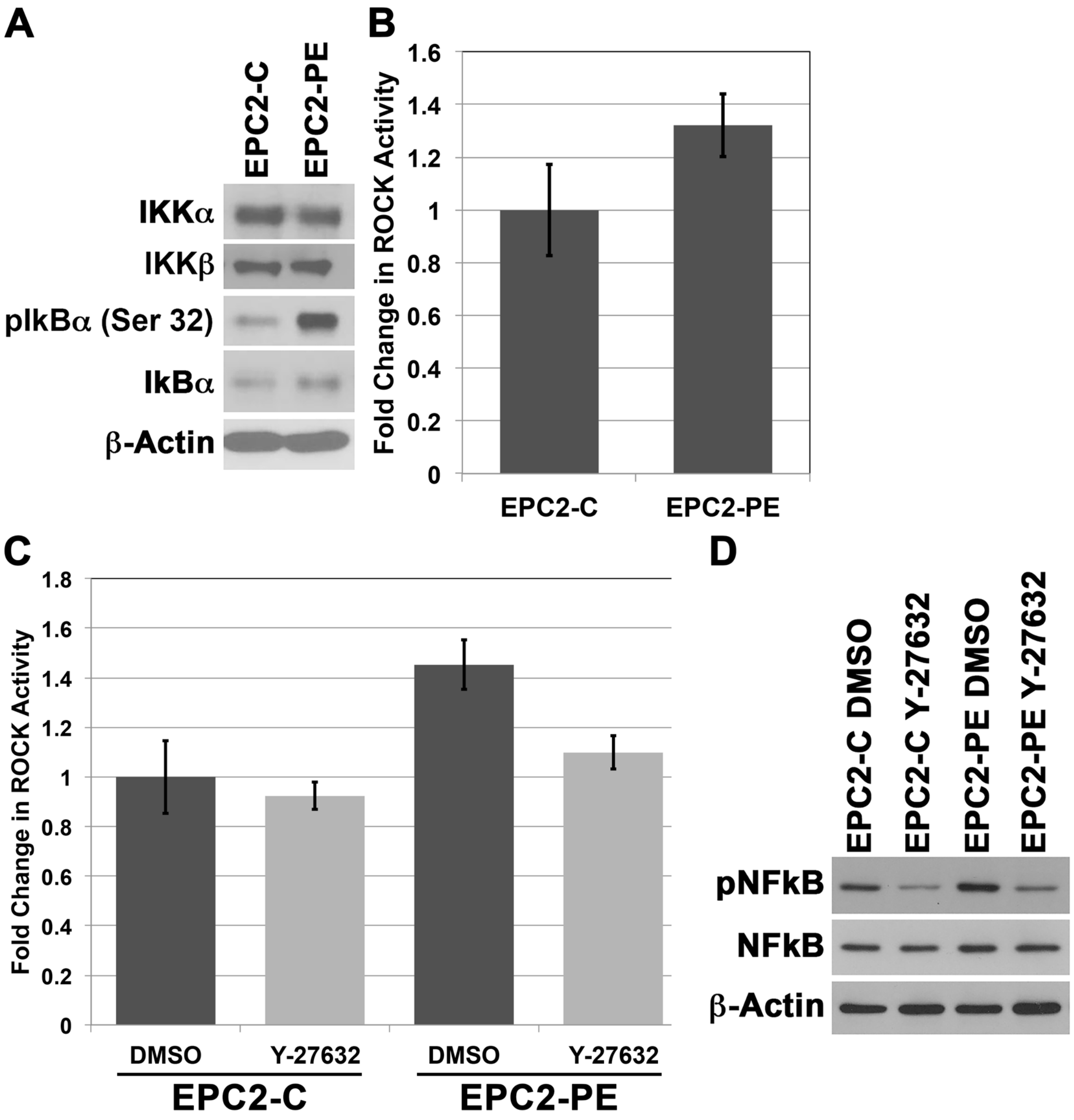

Supplementary Figure 3: The IKK pathway and Rho-kinase activity play a role in the activation of NFkB. **(A)** Western blot analysis of the IKK pathway shows an increase in pIkBα expression. **(B)** Colorimetric *in vitro* ROCK activity assay shows an increase in ROCK activity in EPC2-PE cells. **(C)** Inhibition of ROCK activity with Y-27632 ROCK inhibitor. **(D)** Western blot analysis demonstrates inhibition of pNFkB expression in EPC2 cells treated with Y-27632 ROCK inhibitor.

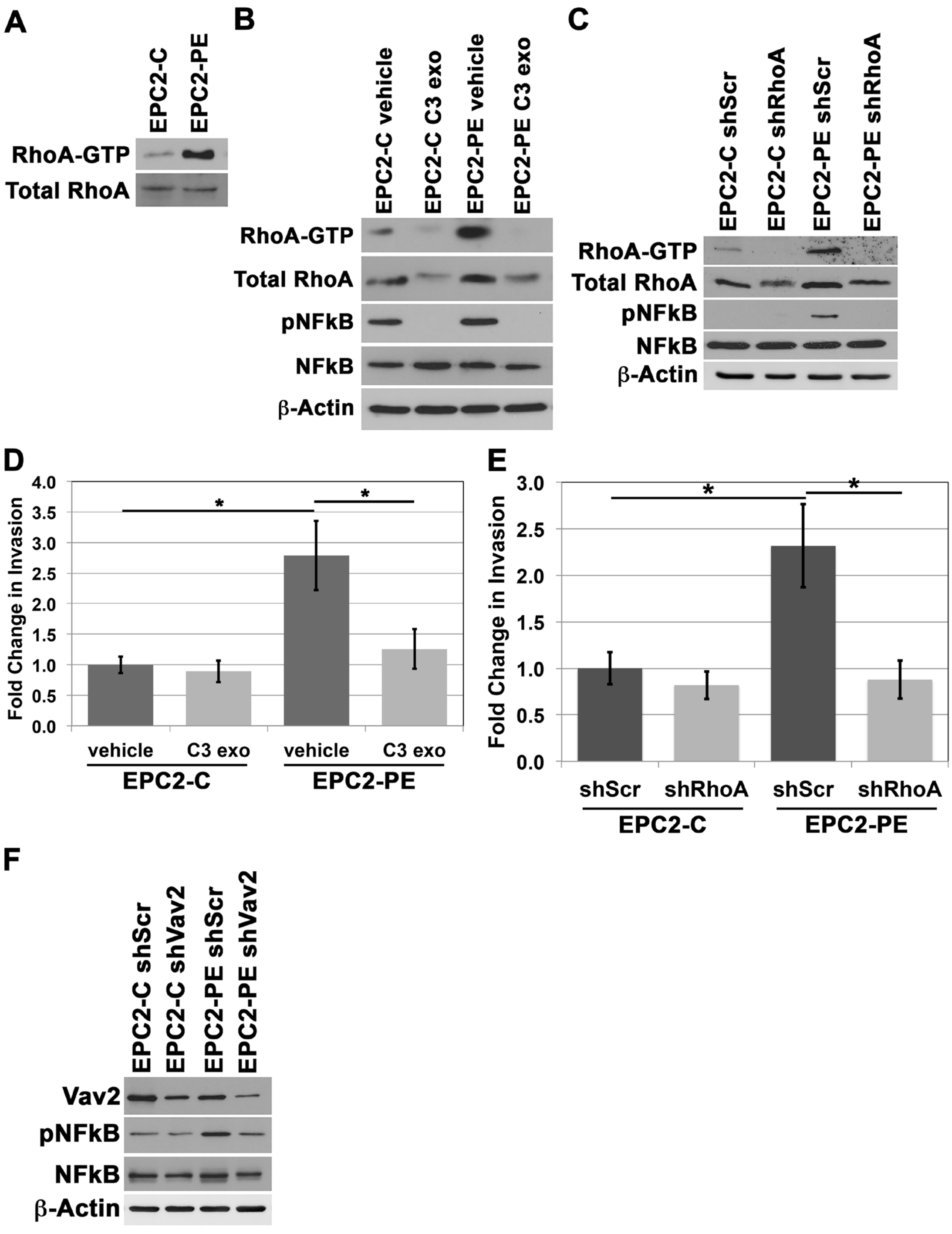

Supplementary Figure 4: RhoA is involved in regulating NFkB activity. **(A)** RhoA pulldown activation assay demonstrates increased RhoA-GTP in EPC2-PE cells. **(B)** Western blot analysis demonstrates inhibition of RhoA-GTP with C3 exotransferase in EPC2-PE cells results in inhibition of pNFkB expression. **(C)** Western blot analysis demonstrates inhibition of RhoA-GTP with shRhoA in EPC2-PE cells results in inhibition of pNFkB expression. **(D)** *In vitro* invasion assays demonstrate a significant decrease in invasion of EPC2-PE cells when RhoA-GTP is inhibited by C3 exotransferase. **(E)** *In vitro* invasion assays demonstrate a significant decrease in invasion of EPC1-PE cells when RhoA-GTP is inhibited by RhoA shRNA. **(F)** Western blot analysis demonstrates inhibition of Vav2 with shVav2 in EPC2-PE cells results in decreased pNFkB expression. * $p<0.05$.

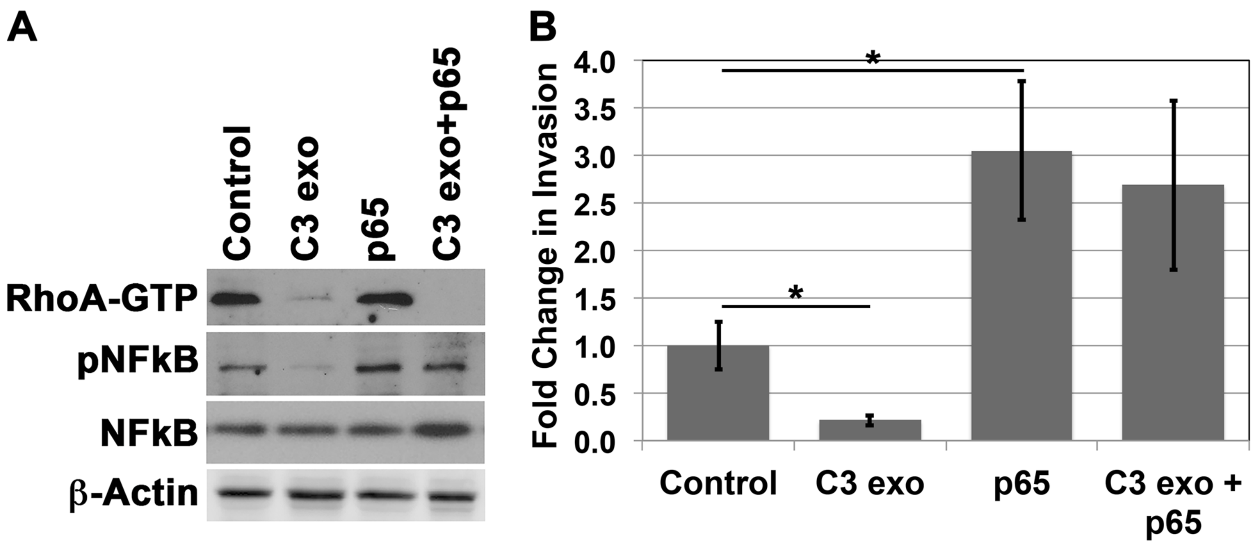

Supplementary Figure 5: NFkB overexpression rescues invasion after RhoA inhibition. **(A)** Western blot analysis demonstrates that treatment of EPC2-PE cells with C3 exotransferase results in inhibition of RhoA and pNFkB, p65 cDNA nucleofection results in increased expression of pNFkB, and C3 exotransferase treatment followed by p65 cDNA nucleofection results in a rescue of pNFkB expression. **(B)** *In vitro* invasion assays demonstrate that treatment of EPC2-PE cells with C3 exotransferase results in inhibition of invasion, p65 cDNA nucleofection results in increased invasion, and C3 exotransferase treatment followed by p65 cDNA nucleofection results in a rescue of the invasive phenotype. * $p<0.05$.
